# Venoarterial ecmo as bridge to heart transplantation. a good chance

**DOI:** 10.1186/2197-425X-3-S1-A906

**Published:** 2015-10-01

**Authors:** R Viejo Moreno, JL Pérez Vela, M Talayero Giménez de Azcárate, MA Corres Peiretti, E Renes Carreño, T Grau Carmona, J Gutiérrez Rodríguez, JC Montejo González

**Affiliations:** 12 de Octubre University Hospital, Cardiologic ICU. Intensive Care Department, Madrid, Spain

## Introduction

Veno-arterial extracorporeal membrane oxigenation (VA-ECMO) is a increasingly device used in patients with a refractory cardiogenic shock. The most important decision before the implantation is to establish the best moment and what is the objective on the basis of INTERMACS criteria.

## Objectives

To analyze our results in the management of refractory cardiogenic shock (CS) which required venoarterial extracorporeal membrane oxygenation (VA-ECMO) as bridge to heart transplantation. Are these results similar to the other transplanted patients without ECMO?

## Methods

An observational prospective study was conducted from March 2010 to February 2015. We reviewed the clinical characteristics and evolution of patients who received venoarterial ECMO as bridge to heart transplant. We analyzed demographic variables, underlying cause of CS, complications and global survival. The quantitative variables are presented as mean ± SD or as median and 25^th^ -75^th^ percentile range and as percentage.

## Results

We studied 17 patients. The mean age was 40 ± 13 years, 56% female. Most common underlying causes were idiopathic dilated cardiomyopathy (41.2%), ischemic dilated cardiomyopathy (23.5%) and acute myocardial infarction (11.7%). Before insertion, all patients were under inotropic support and 75% had intra aortic balloon pump. Renal failure was in 31% of patients.

All device insertions were peripheral, 88% femorofemoral. The length of ECMO was 192 hours (129 - 317) with a maximum of 480 hours. Only one patient presented several bleeding due to accidental decannulation and other patient had a several vascular complication (lower extremity ischemia). Nine patients (53%) had renal failure and four of them developed multiple organ failure. Fourteen of seventeen patients received a heart transplant (82%). It was possible a successful weaning in ten cases but two patients presented primary graft dysfunction and needed a second VA-ECMO. Both could be weaned from ECMO. Global survival in our series was 70.6%. Overall survival in the series analyzed was around 80-85%.

## Conclusions

VA-ECMO should be considered an effective therapeutic support for patients with refractory CS because it enables patients to reach to transplantation with favorable results. These are slightly lower than the results reported in the general series of heart transplant.

